# Surgical Written Consent in Aesthetic Plastic Surgery: A Plastic Center Audit of Surgical Consent Standards

**DOI:** 10.7759/cureus.51701

**Published:** 2024-01-05

**Authors:** Mohamed B Ahmed, Fatima S Almohannadi, Bara A Shraim, Ghanem Aljassem, Salim Al-lahham, Abeer Alsherawi

**Affiliations:** 1 Plastic and Reconstructive Surgery, Qatar University, Doha, QAT; 2 Plastic and Reconstructive Surgery, Hamad Medical Corporation, Doha, QAT

**Keywords:** american society of plastic surgeons, indemnity risk, ethical and legal principles in medical practice, surgical informed consent, audit, complication, quality, plastic surgery, informed consent

## Abstract

Background

Informed consent is a fundamental aspect of modern medical practice that requires effective communication and an ample understanding of medical ethics, patient autonomy, and the legal obligations of healthcare professionals. Maintaining high-quality surgical informed consent is a crucial step in the healthcare process. Thus, we aimed to obtain surgical written consent on abdominoplasty, suction-assisted abdominal dermo lipectomy, and lower body lifting from our hospital database over three months (January to March, 2023) to assess our level across the American Society of Plastic Surgeons (ASPS) system.

Results

A total of 45 surgical written consents were obtained and 37 consents remained after exclusion. Bleeding, infection, and hematoma were mentioned in more than 80% of our consents. However, important complications such as ileus and umbilical malposition were never mentioned. Overall, the completion rate of written complications ranged from 14% to 56% in comparison to data from the ASPS.

Conclusions

Our results show a measurable inconsistency in surgical written consents at our center. Thus, establishing a comprehensive and accurate surgical written consent will aid in protecting our center against deficient surgical written consent accusations, improve the experience of patients, and enhance the quality of service provided by our center.

## Introduction

Clear and comprehensive informed consent in surgery is a cornerstone in the process of a patient’s experience in any surgical department [1]. It is critical to include a sizeable volume of medical information yet convey it in a patient-friendly simplified manner. This process allows the patient to make an informed decision to undergo a specific surgical procedure. Nonetheless, it is essential to preserve complete documentation of the consent in the patient’s notes. The significance of informed consent increases in the aesthetic plastic surgery field because we are dealing with healthy individuals who mostly have no indication for surgery, as well as patients with body dysmorphic disorder, who might never be satisfied [2,3].

Therefore, it is also important to understand the definition of informed consent. It is the involvement of the patient as a participant in the decision-making of a medical or surgical procedure, after a clear understanding of the procedure and its related medical information and more importantly the risks that might be involved [4,5]. Legally, the patient should be mentally stable and not under the influence of alcohol and/or drugs. Therefore, to give valid consent, the patient must be able to comprehend the provided information about the surgical procedure [6]. A valid informed consent requires achieving seven criteria, including (i) there must be ability to understand and make decisions, (ii) the decision-making must be voluntary, (iii) all essential information must be provided, (iv) a recommended plan must be provided, (v) clear understanding of the terms must be there, (iv) there has to be a decision regarding the plan, and (vii) the plan must be authorized. Only when all these criteria are achieved, can informed consent be said to have been given by the patient. In case all criteria are achieved, but the patient rejects the plan, then it is considered an informed refusal [6].

Maintaining a high standard of surgical written consent is a very demanding yet important task in the surgical field generally and in aesthetic plastic surgery practice specifically. Thus, in this study, we aim to assess the quality of our center's surgical written consents and shed light on the gaps in order to improve them.

## Materials and methods

The study spanned three months, from January to March 2023, focusing on the acquisition of surgical consent related to Abdominoplasty, Suction Assisted Abdominal Dermo-lipectomy (SADL), and Lower Body Lifting (LBL). These consents were obtained based on the corresponding procedure codes within our health center system. Data was meticulously reviewed and extracted from these consents by two authors (FA and BS). In instances of discrepancies, a third author (MB) was consulted to arbitrate and resolve conflicts. Consents involving combined procedures, such as those involving both the trunk and breasts, were excluded from the analysis to ensure the clarity and specificity of the findings. The study was conducted at the Surgical Specialty Center, Hamad Medical Corporation, Doha, Qatar.

The collected data encompassed crucial information such as patient demographics (including name, date of birth, and hospital identification number), surgeon details (encompassing name, signature, date, and role), the procedure's name as documented on the consent, a list of potential complications, and the availability of an Arabic translation if the patient identified as Arab. Furthermore, a thorough examination of the complications listed for each surgery was conducted, comparing them with the 2016 version of the American Society of Plastic Surgeons (ASPS) complications list [2].

Subsequently, comprehensive statistical analyses were performed to evaluate the completion rates of complications lists for each consent in comparison to the ASPS reference list. The study also scrutinized the availability of patients' demographic details, the inclusion of surgeon information, and the presence of an Arabic translation when applicable.

## Results

A total of 45 consents were recruited over January-March 2023 for the three selected procedures: Abdominoplasty, SADL, and LBL. Eight consents were excluded because of the presence of trunk and breast surgeries in the same consent. Thus, 37 consents (four LBL, 27 SADL, and six Abdominoplasty) were eligible for the analyses. The total number of complications in the ASPS consent form is as follows: 29 for Abdominoplasty, 30 for LBL, and 32 for SADL surgeries. The analyses’ results showed huge variations (Table 1).

**Table 1:** Descriptive results of the included surgical consents that show the rate of presence/absence of ASPS complications list for each procedure. ASPS: American Society of Plastic Surgeons; n: number of consents

Type of procedure	Presence of the complication	Lower body lift (n=4)	Suction-assisted abdominal dermo-lipectomy (n=27)	Abdominoplasty (n=6)
Bleeding	Absent	0 (0%)	0 (0%)	1 (16.67%)
	Present	4 (100%)	27 (100.00%)	5 (83.33%)
Infection	Present	4 (100%)	27 (100%)	6 (100%)
Hematoma	Absent	0 (0%)	2 (7.41%)	0 (0%)
	Present	4 (100%)	25 (92.59%)	6 (100%)
Seroma	Absent	0 (0%)	1 (3.70%)	2 (33.33%)
	Present	4 (100%)	26 (96.30%)	4 (66.67%)
Possible hernia repair	Absent	4 (100%)	27 (100%)	6 (100%)
Injury of deep structures	Absent	2 (50%)	13 (48.15%)	3 (50%)
	Present	2 (50%)	14 (51.85%)	3 (50%)
Ileus	Absent	4 (100%)	27 (100%)	6 (100%)
Sensory changes	Absent	3 (75%)	23 (85.19%)	6 (100%)
	Present	1 (25%)	4 (14.81%)	0 (0.00%)
Asymmetry	Absent	1 (25%)	9 (33.33%)	5 (83.33%)
	Present	3 (75%)	18 (66.67%)	1 (16.67%)
Malposition of umbilicus	Absent	4 (100%)	27 (100%)	6 (100%)
Fat necrosis	Absent	4 (100%)	21 (77.78%)	6 (100%)
	Present	0 (0%)	6 (22.22%)	0 (0%)
Pubic distortion	Absent	4 (100%)	27 (100%)	6 (100%)
Hypertrophic scar or keloid	Absent	1 (25%)	4 (14.81%)	1 (16.67%)
	Present	3 (75%)	23 (85.19%)	5 (83.33%)
Skin necrosis	Absent	3 (75%)	9 (33.33%)	6 (100%)
	Present	1 (25%)	18 (66.67%)	0 (0%)
Delayed healing	Absent	4 (100%)	26 (96.30%)	6 (100%)
	Present	0 (0.00%)	1 (3.70%)	0 (0%)
Revision surgery	Absent	0 (0.00%)	11 (40.74%)	2 (33.33%)
	Present	4 (100%)	16 (59.26%)	4 (66.67%)
Pain	Absent	0 (0%)	20 (74.07%)	1 (16.67%)
	Present	4 (100%)	7 (25.93%)	5 (83.33%)
Allergic reactions	Absent	3 (75%)	19 (70.37%)	3 (50%)
	Present	1 (25%)	8 (29.63%)	3 (50%)
Fat or air embolism	Absent	4 (100%)	22 (81.48%)	6 (100%)
	Present	0 (0.00%)	5 (18.52%)	0 (0%)
Lymphedema	Absent	4 (100%)	27 (100%)	6 (100%)
Unsatisfactory results	Absent	1 (25%)	14 (51.85%)	4 (66.67%)
	Present	3 (75%)	13 (48.15%)	2 (33.33%)
Stitch sinus	Absent	4 (100%)	26 (96.30%)	6 (100%)
	Present	0 (0%)	1 (3.70%)	0 (0%)
Wound dehiscence	Absent	0 (0%)	5 (18.52%)	2 (33.33%)
	Present	4 (100%)	22 (81.48%)	4 (66.67%)
Contour irregularities	Absent	4 (100%)	24 (88.89%)	6 (100%)
	Present	0 (0%)	3 (11.11%)	0 (0%)
Reloosening of skin	Absent	4 (100%)	27 (100%)	6 (100%)
Adhesions	Absent	4 (100%)	27 (100%)	6 (100%)
VTE	Absent	3 (75%)	24 (88.89%)	6 (100%)
	Present	1 (25%)	3 (11.11%)	0 (0%)
Cardiopulmonary complications	Absent	3 (75%)	26 (96.30%)	6 (100%)
	Present	1 (25%)	1 (3.70%)	0 (0%)
Shock	Absent	4 (100%)	27 (100%)	6 (100%)
Burn	Absent		18 (66.67%)	
	Present		9 (33.33%)	
Unknown risk	Absent		27 (100%)	
Cannula fragmentation	Absent		27 (100%)	
Flattening of the buttocks	Absent	4 (100%)		

A few complications were present in more than 80% of the consents such as bleeding, infection, and hematoma. On the other hand, many complications were never mentioned in any consent such as Ileus, malposition of the umbilicus, pubic distortion, lymphedema, adhesions, and shock (Table 1). In addition, important and expected complications like sensory changes, injury to the deep structures, and burns were found to be present with a very low rate in the consents. 

Patient demographics and surgeon’s details were present in all the consents (Table 2). Furthermore, Arabic translation for Arab patients was available in more than 70% of the consents and three patients were non-Arab (English speakers) (Table 3). Across all the consents, the completion rate ranged between around 14-56% when compared to the ASPS complications list (Table 4). Furthermore, a few observations were made during data collection like unclear handwriting in some consents and a few spelling mistakes too. In addition, a couple of extra complications (not mentioned in the ASPS list) were mentioned such as ecchymosis, transfusion complications, pigmentation, and reaction to anesthesia drugs. It was noted that in a few consents, there was inconsistency between the operation mentioned in the consent and the one mentioned in the operative note.

**Table 2:** Patient demographics and surgeon’s details completion rate.

Factors	Percentage (%)
Patient's name	100
Date of birth	100
Hospital identifier	100
Surgeon’s name	100
Signature and date	100
Level	100

**Table 3:** Arabic translation availability rate.

Arabic translation	Frequency (n)	Percentage (%)
Absent	10	29.41
Present	24	70.59
Total	34	100

**Table 4:** Descriptive results of the included surgical consent total completion rate N: number of consents

	N	Mean	Standard deviation	Min	Max
Completion rate %	37	34.06	9.04	13.79	56.25

## Discussion

Given the importance of the surgical written consent quality, this audit was carried out to evaluate the standards of consent mainly in terms of the complications list for aesthetic surgeries in our center. The process of consent taking in our center includes manually filling a hard copy of a printed form with the name of the procedure along with the possible complications. This process is usually done the day before or on the day of surgery. Our results showed inconsistent standards among our center consents as well as huge variation when compared to ASPS consents, concluding that the quality of the existing surgical consent process in our center is suboptimal.

This might be due to several reasons including differences in the surgical background among plastic surgeons, language barrier, lack of standardized template or unified prewritten consent, and lack of allocated time for writing and discussing the consent. Having prewritten consents as proposed by the ASPS ensures that surgeons don’t miss to mention any possible complication and ultimately reduces the amount of time required to fill the form and allows more time for a proper consent-taking process. In addition, surgical consents are mainly written by junior doctors (residents) and this might lead to low-quality consents. Thus, efforts and audits have been performed worldwide in the clinical field to overcome the obstacles and challenges of obtaining well-informed consent. We have noticed that this outcome might be due to the lack of direct exposure of the junior resident who is taking the consent to such aesthetic procedures, and sometimes a shortage of time spent with each patient to complete a surgical written consent, thus obtaining insufficient consent [7].

Another audit had similar findings when they assessed the process of obtaining consent for trauma cases in an Orthoplastic Hand Unit [8]. The results of the audit were able to identify a few gaps in the junior doctors' consenting process; thus, a plan was made to integrate the optimal consent process into the department’s orientation program delivered to the junior doctors. Other than enhancing the quality of consent taking, patients should be given enough time to think about the procedure and its possible complications. Taking consent on the day of surgery or a day before deprives patients of their right to consider and ask detailed questions about their surgery. In addition, one article mentions that patients usually have selective hearing when they feel rushed or pressured [9]. This puts patients and surgeons at a high risk of dissatisfaction and malpractice claims.

Another point that might restrict our residents from explaining all the major risks and complications is that patients might get overwhelmed emotionally and overloaded with information that could stress them even further. This requires the surgeon to convey the information in a simplified and clear context. Several techniques might help to overcome such obstacles like allowing the patient’s social support system such as family and loved ones to be present with him/her and giving the patient more time to digest the load of information explained while consenting. Additionally, providing patients with a copy of the surgical written consent might help them to understand the scheduled procedure clearly and reflect on it [10]. Also, it is useful to have available handouts of normal anatomy or figures of realistic expectations of procedures at the clinic [9]. This might help patients understand the impact of the surgery on their body.

Our results showed that few complications were written in the majority of consents while the majority of complications were absent in many. In addition, a few important complications like reloosening of skin, lymphedema, and adhesions were never written in any consent. Lack of complication listing was also shown in a study of negligence claims and complaints, where complications were not mentioned or fully explained in 70% of the cases [11]. This might predispose surgeons to malpractice claims related to inadequate surgical written consent once an unlisted complication takes place. Furthermore, it has been previously demonstrated that one of the main causes of why patients sue their surgeons is because of deficiencies in informed consent [12]. As a result, insurance companies might issue lawsuits and it might end up with an indemnity payment. This was highlighted in a published review that discussed the medico-legal aspects of informed surgical consent in orthopedic surgery [13]. The review identified 28 lawsuits with a claim of inadequate informed consent; 13 patients out of the 28 developed a complication that was not written in the consent and seven of the 13 cases did not result in any indemnity payment. In another study, legal outcomes of having inadequate consent included having 24 out of 34 malpractice claims to be settled with payment [11]. Thus, writing adequate surgical written consent with an accurate complications list might help in reducing the allegations of inadequate informed consent and therefore the indemnity risk.

To overcome the aforementioned gaps, we will propose to adopt a unified structured prewritten surgical written consent recommended by ASPS to be used in our center initially. In addition, integrating the main principles of proper surgical written consent in the educational program of the residents along with establishing a reliable and comprehensive source as a reference for the junior residents will improve consent quality dramatically. Equally important, review and supervision by senior surgeons of the consents written by junior residents will help in reducing deficiencies and educating and guiding them to ensure having high quality and consistent surgical written consent. Furthermore, we plan to repeat this audit annually with the above recommendations taken into consideration in order to assess our progress and ensure continuous improvement. 

Our focus in this audit was directed mainly to the technical aspect of the consent process; however, we plan to widen our research scope in the future and assess patients’ feedback and perspectives on the process and whether this approach is efficient and sufficient or not.

## Conclusions

A consistent and organized approach to obtaining surgical written consent is a crucial step and ought to be implemented in all healthcare systems with similar quality levels. Therefore, huge efforts and multiple audits have been carried out globally to assess and address the barriers and difficulties associated with obtaining proper surgical written consent. In our audit, we were able to identify gaps in our center's surgical written consent process and propose solutions for them. The inability to fix these gaps might compromise our center's quality of care and lead to legal consequences.
